# Identification of the single and combined acute toxicity of Cr and Ni with *Heterocypris* sp. and the quantitative structure-activity relationship (QSAR) model

**DOI:** 10.1371/journal.pone.0300800

**Published:** 2024-03-21

**Authors:** Chi Su, Yilong Hua, Yi Liu, Shu Tao, Fei Jia, Wenhui Zhao, Wangyang Lin

**Affiliations:** 1 School of Resources Environment and Safety Engineering, University of South China, Hengyang, China; 2 School of Mathematics and Physics, University of South China, Hengyang, China; 3 School of Civil Engineering, University of South China, Hengyang, China; 4 Shanxi Province Changzhi City Wuxiang County Jia Huo Township People’s Government, Changzhi, China; 5 College of Mechanical Engineering, University of South China, Hengyang, China; University of the Punjab, PAKISTAN

## Abstract

Mining wastewater with heavy metals poses a serious threat to the ecological environment. However, the acute single and combined ecological effects of heavy metals, such as chromium (Cr) and nickel (Ni), on freshwater ostracods, and the development of relevant prediction models, remain poorly understood. In this study, *Heterocypris* sp. was chosen to investigate the single and combined acute toxicity of Cr and Ni. Then, the quantitative structure-activity relationship (QSAR) model was used to predict the combined toxicity of Cr and Ni. The single acute toxicity experiments revealed high toxicity for both Cr and Ni. In addition, Cr exhibited greater toxicity compared to Ni, as evidenced by its lower 96-hour half-lethal concentration (LC_50_) of 1.07 mg/L compared to 4.7 mg/L for Ni. Furthermore, the combined acute toxicity experiments showed that the toxicity of Cr-Ni was higher than Ni but lower than Cr. Compared with the concentration addition (CA) and independent action (IA) models, the predicted results of the QSAR model were more consistent with the experimental results for the Cr-Ni combined acute toxicity. So, the high accuracy of QSAR model identified its feasibility to predict the toxicity of heavy metal pollutants in mining wastewater.

## 1. Introduction

Mine exploitation can provide abundant resources, but the serious harm caused by a large number of toxic substances, such as heavy metals, to aquatic ecosystems and human health has attracted widespread attention to the heavy metal pollution of mining water environments. Physical and chemical monitoring is the most common method for evaluating water environmental health [1]. However, this method has obvious limitations. First, it does not take into account the ability of environmental organisms to absorb and use pollutants. Second, it does not assess the toxic effects of pollutants such as heavy metals on the surrounding ecosystem [2]. Biological monitoring uses specific biological indicators to characterize the degree of pollution. This approach is more realistic and comprehensive [3]. Therefore, it is essential and urgent to assess the ecological toxicity effects of heavy metal pollution in water bodies on aquatic organisms.

Chromium (Cr) and nickel (Ni) are common heavy metal pollutants in mine wastewater, which can both be harmful to the activity of aquatic organisms and human health through processes such as bioaccumulation [4]. Aliya et al. [5] investigated the pollution levels of Cr and Ni in the soils of the Sukinda mining area using the geoaccumulation index method (Cr: 2 and Ni: 5). The results showed that the mining area was severely to extremely polluted, which seriously affected the health of local residents. However, single pollution situations of Cr and Ni are almost nonexistent in the real environment [6]. Cr and Ni typically coexist in mixed forms, and the interaction between Cr and Ni can affect the strength of their combined toxicity [7]. Liu et al. [8] studied that pollutants can exhibit three types of toxicity effects when acting in combination: synergy, antagonism, and summation. Toxicity experiments are important tools for assessing the impacts of various toxins on ecosystems and are widely used for determining specific biological indicators and understanding the health status of ecosystems [9]. Conducting toxicity experiments using heavy metal elements is fundamental to understanding their toxicity to organisms and is also necessary for the safety assessment of ecotoxicology.

Freshwater ostracods belong to the class crustacea within the phylum arthropoda. Freshwater ostracods are small and highly sensitive to pollutants, making them suitable for laboratory cultivation. They have been widely used in the study of the toxicity of single or mixed heavy metals (ppb-ppm) in water [10–12], soil [13–16], and sediments [17, 18]. Zhang et al. [19] investigated the soil heavy metal pollution in a uranium mine in South China, using *Cypridopsis vidua* and *Heterocypris* sp. to assess the soil ecological toxicity. The results showed that the higher the heavy metal concentration, the higher the mortality of the organisms. Gonzalez-Regalado et al. [20] investigated the impact of heavy metal pollution in estuaries on the ostracods communities along the southwest coast of Spain. The study found that the diversity of ostracods was significantly decreased by heavy metal pollutants. Ruiz et al. [21] collected 55 ostracods samples from the coastal area of Huelva, southwest Spain. The study revealed that heavy metal pollution in some river reaches led to a decrease in ostracod abundance.

Biotoxic effects can also be predicted through numerical models, and some theoretical models have been applied in practice [22–24]. The Quantitative Structure Activity Relationship (QSAR) model is often used in toxicology to predict the physicochemical properties and toxicological effects of heavy metals or heavy metal compounds based on their characteristic parameters [25]. Wang et al. [26] used hydrophobicity descriptors to establish a QSAR model to predict the toxicity of perfluorocarbonic acid to luminescent bacteria. The regression coefficient was high, and the model predicted well. Song et al. [27] studied the acute toxicity of six naphthoquinone compounds to *Daphnia magna* and used a QSAR model to predict the results. The predictions were relatively reliable.

Ostracods are a novel and effective environmental indicator organism. However, research on ostracods is mainly focused on taxonomy [28], with fewer studies conducted in ecotoxicology. Therefore, this study conducted single and combined acute toxicity experiments of the common pollutants Cr and Ni on the test organism *Heterocypris* sp., analyzing its sensitivity. QSAR models can be used for joint toxicity prediction. Combined with toxicity experimental results, the relationship between toxicity and concentration can be observed. Currently, QSAR models are mainly used for toxicity prediction of fish and daphnia [29], and less for ostracod toxicity prediction. Therefore, this study applies QSAR models to predict toxicity in ostracods, providing new insights for research in this field and enhancing the application level of QSAR models in this area.

## 2. Materials and methods

No specific permissions were required for the described field studies. We confirmed that the field studies did not involve endangered or protected species.

### 2.1 Materials

Potassium dichromate (K_2_Cr_2_O_7_, analytical grade) and nickel nitrate (Ni(NO_3_)_2_, analytical grade) were separately prepared into 100 mg/L stock solutions using deionized water. The stock solutions were then diluted with aerated water to the experimental concentrations (Cr: 0.2–15 mg/L; Ni: 3–44 mg/L). The experimental concentration setting was based on the minimum non-lethal concentration and the maximum lethal concentration.

The test organisms, *Heterocypris* sp., were collected from lakes and rivers around Hengyang City, Hunan Province. They were identified and classified based on their soft and hard body characteristics. The organisms were cultured in a laboratory incubator for 180 days under conditions suitable for ostracod survival [30]: 25°C, a 24-hour light/dark cycle of 2:1, pH 7.5 ± 0.2, and dissolved oxygen > 5 mg/L. Natural water was added regularly, and protein chlorella (concentration: 500 g/L) was fed to the organisms. Feeding was stopped 24 hours before the start of the experiment to ensure the stability of the toxicological biomaterials. In order to maintain consistency, adult ostracods of similar size and vitality were selected for the experiment.

### 2.2 Experiments and methods

#### 2.2.1 Cr and Ni single acute toxicity analysis

Single polarity toxicity analysis experiments were conducted with 10 specimens per group, using a static water (50 mL) exposure method for a period of 96 hours (no feeding during exposure) [31]. Different Cr and Ni concentration levels (Table 1) and a blank control group without heavy metals were set up, and each group of experiments was repeated three times. During the experiment, the number of deaths of ostracods in each experimental group was observed and recorded at 24, 48, 72, and 96 hours, and a concentration-response curve (CRC) was drawn. The temperature, pH, hardness, and dissolved oxygen content of the solution were also measured before and after the experiment.

**Table 1:** Design values of the single acute toxicity experimental concentrations of *Heterocypris* sp.

Group	K_2_Cr_2_O_7_ mg/L	Ni(NO_3_)_2_ mg/L
1	0.2	3
2	0.59	5.87
3	1.73	11.49
4	5.1	22.48
5	15	44

#### 2.2.2 Cr-Ni combined acute toxicity analysis

**Fig 1:**
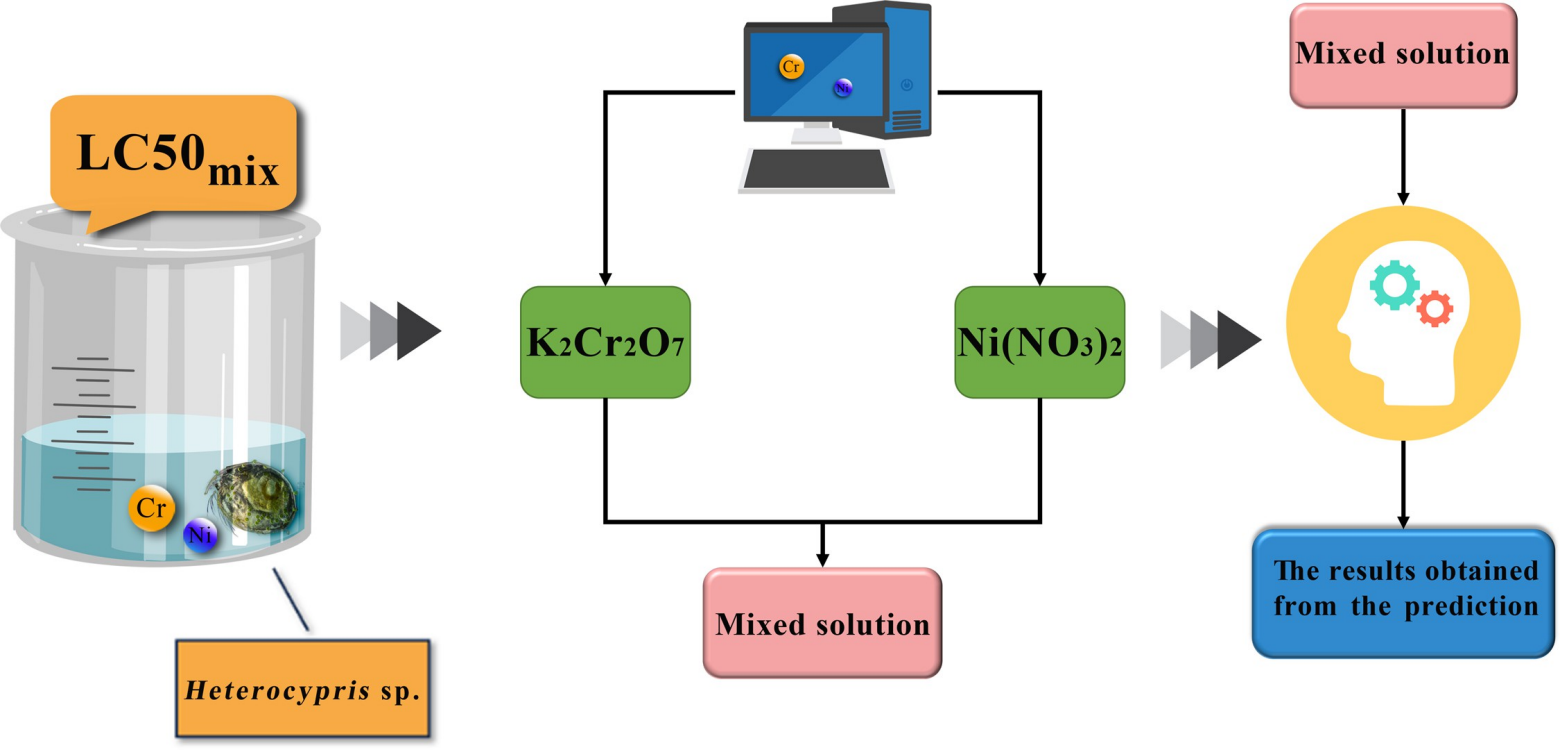
Combined acute toxicity experiment flowchart.

**Fig 2:**
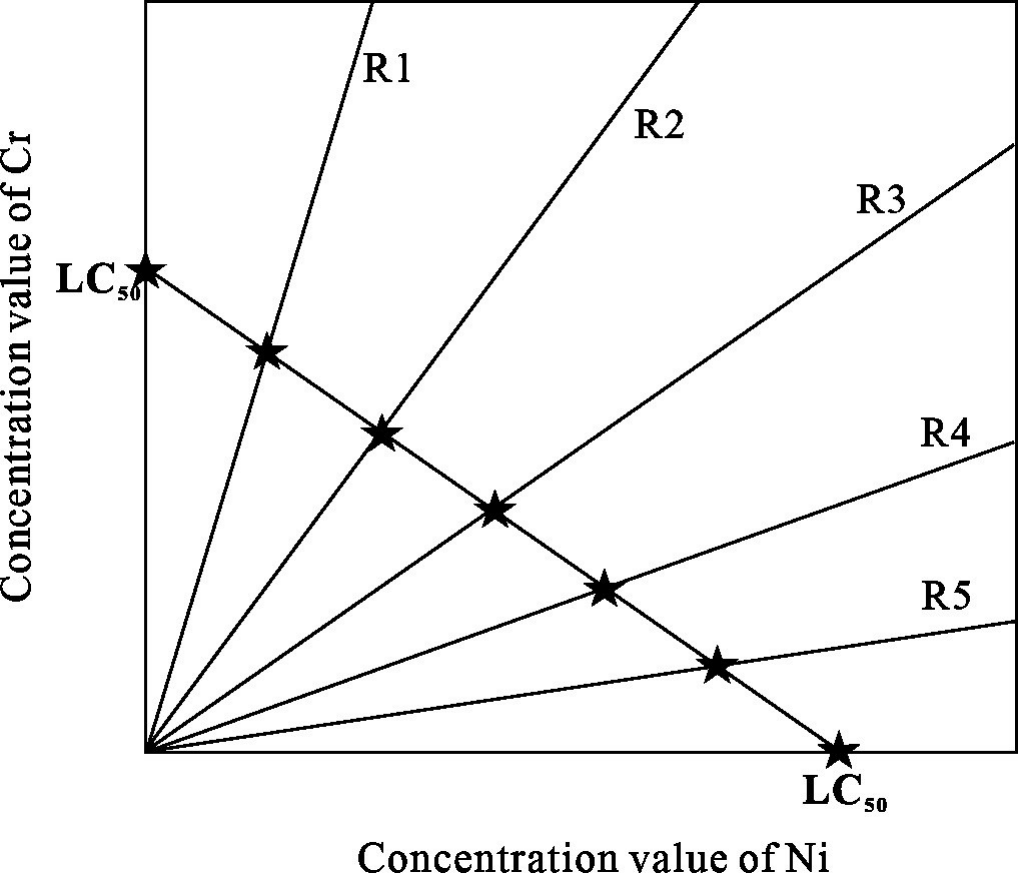
Principle diagram of the direct equipartition ray method.

The combined acute toxicity analysis was conducted based on the lethal concentrations obtained from single acute toxicity experiments. The 4-day direct exposure acute toxicity test method was employed under static exposure conditions (Fig 1). Experimental concentrations were designed using the equivalent effect concentration ratio (EECR) method and the direct equipartition ray (EquRay) method (Table 2). Both methods can be used to assess the mixture toxicity of component mixing ratios and concentration levels in binary systems. EECR is based on the condition that the effect concentrations of all components in the mixture reach the same effect concentration, forming a series of component concentration ratios. Then this method analyzes the pattern of changes in combined acute toxicity [32–34]. The EECR method utilized the 96 h-LC_10_, LC_30_, and LC_50_ values of Cr and Ni for *Heterocypris* sp. obtained from single acute toxicity experiments. Three groups of Cr-Ni concentration ratios (such as EECR 10, EECR 30, and EECR 50) were constructed based on the equivalent effect fixed concentration ratio method. For example, EECR 50 represents the sum of the 96 h-LC_50_ values of Cr and Ni. The EquRay method identifies representative concentration points within the mixture and designs concentrations for various mixtures to assess how toxicity changes across these combinations. This method employs a rational and effective selection process for these representative points [35]. The EquRay method employs the representative lethal concentrations (LC_50_) of Cr and Ni as the coordinates on the x-axis and y-axis, respectively (Fig 2). Connecting the reference points forms line segments, and these line segments are divided into five equidistant points (EquRay 1 (R1) ‐ EquRay 5 (R5)). For example, R1 represents the sum of 1/6 of the 96 h-LC_50_ value of Cr and 5/6 of the 96 h-LC_50_ value of Ni, and R2-R5 follow a similar progression. To analyze the toxicity process of binary mixtures using the aforementioned methods, the Fixed Ratio Ray Design (FRRD) [36, 37] was employed. This approach involved diluting the mixtures from high to low concentrations while maintaining the established concentration ratios for each group. This resulted in 12 concentrations for experimentation.

**Table 2:** Design of concentration ratios for the combined acute toxicity experiments of *Heterocypris* sp.

Concentration design	Concentration ratio	
	K_2_Cr_2_O_7_	Ni(NO_3_)_2_
EECR 10	0.05	0.95
EECR 30	0.08	0.92
EECR 50	0.11	0.89
EquRay 1	0.53	0.47
EquRay 2	0.31	0.69
EquRay 3	0.19	0.81
EquRay 4	0.10	0.9
EquRay 5	0.04	0.96

### 2.3 Qualitative evaluation method for Cr-Ni combined toxicity

The toxicity evaluation uses Synergistic Ratio (SR) to measure the combined toxicity, and the calculation formula for SR value is as follows [38]:

$$SR=\frac{C_{i}}{E_{i}}$$ (1)

In the formula, *C*_*i*_ represents the single heavy metal 96 h-LC_50_ value, and *E*_*i*_ represents the combined heavy metal 96 h-LC_50_ value. When *SR* = 1, the toxicity is considered an additive effect. when *SR*<1, it is an antagonistic effect, and when *SR*>1, it is a synergistic effect.

### 2.4 The prediction model of QSAR

**Fig 3:**
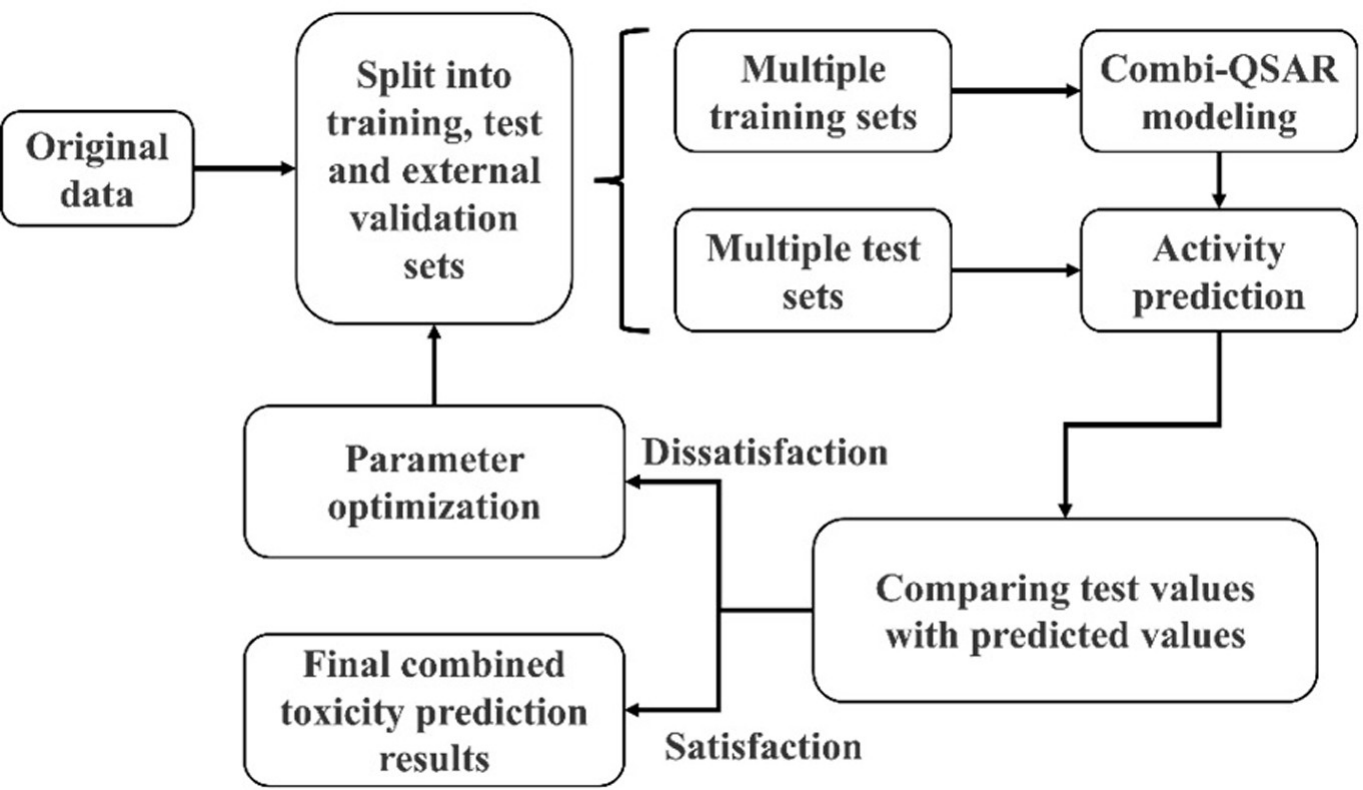
QSAR prediction model flowchart.

Predicting QSAR models based on the results of combined acute toxicity experiments [39]. The QSAR model divided the original data into an 8:2 training and test set for model development and validation, respectively. The model selected the dataset with the highest predictive accuracy as the final result (Fig 3) [40].

The purpose of mathematical statistics is to extract information from data and build predictive models using a variety of statistical techniques.

Ridge regression is a biased estimation regression method specialized for the analysis of collinear data. It sacrifices some information loss and reduces accuracy to obtain regression coefficients that are more practical and reliable. Its results are more accurate than those of the least squares regression method [41].

Extreme Gradient Boosting (XGBoost) is an extension of ensemble algorithms that reduces the error in regression results [42].

Multilayer Perceptron Regression (MLP Regressor) falls within the realm of Artificial Neural Networks (ANN) and is an extension of Backpropagation Neural Networks (BPNN). This algorithm is a popular neural network architecture in toxicology, providing good regression results [43].

Multilinear Regression (MLR) aims to fit a linear model that minimizes the differences or errors between predicted values and actual values [44].

### 2.5 The prediction model of the Concentration addition and the independent action

Concentration addition (CA) and independent action (IA) models are two commonly used combined toxicity prediction models. Their main difference lies in the different ways they handle the interactions between the components in the mixture.

The CA model assumes that the toxic effects of the components in a mixture are additive, and does not consider their interactions. It is typically used for mixtures with similar toxicity mechanisms [45]. The results of the CA model are calculated based on parameters such as the LC_50_ values of the individual components.


$$CA=\sum_{i=1}^{n}C_{i}\times {\left(LC_{50}\right)}_{i}$$ (2)

In the formula, *C*_*i*_ represents the concentration of the first chemical substance in the mixture. (*LC*_50_)_*i*_ represents the half-lethal concentration of the chemical substance.

The IA model assumes that the toxic effects of the components in a mixture are independent, meaning that each chemical can produce an independent effect, without being affected by the other components. It is typically used for mixtures of chemicals with different mechanisms of toxicity [46]. The results of the IA model are also calculated using parameters such as the LC_50_ values of the individual components.


$$IA=1-\prod_{i=1}^{n}\left(1-f_{i}\right)$$ (3)

In the formula, *f*_*i*_ represents the effect fraction of the first chemical substance, indicating the extent of its influence on the organism.

### 2.6 Data processing methods

The MIXTOX toolkit [47] was used to fit the 24, 48, 72, and 96 h CRC data for single toxicity experiments of Cr and Ni, respectively, to obtain the lethal concentrations x (LCx). The QSAR model [48] was used to predict the combined toxicity experiments of Cr and Ni. Three sigmoidal nonlinear functions (Table 3) were selected for fitting based on the concentration-response data of this experiment, and the best fit was selected. The quality of the model was evaluated using the coefficient of determination (R^2^), root mean square error (MSE), or mean absolute error (MAE) [49].

**Table 3:** 3 S-type nonlinear functions and their corresponding inverse functions.

Equation Name	Function	Inverse function
Weibull	*f*(*x*) = 1-exp(-exp(*α*+*β*lg(*x*)))	$x=10^{\left(\mathrm{lg}(-\mathrm{lg}(1-Y)/\mathrm{lg}e)/\mathrm{lg}{\text{g}}_{e-a}\right)/\beta }$
Box-Cox-Weibull(BCW)	*f*(*x*) = 1-exp(-exp(*α*+*β*((*x*^*γ*^-1)/γ)))	$x={\left((\gamma /\beta )\left(\mathrm{lg}(-\mathrm{lg}(1-Y)/\mathrm{lg}e)/{\mathrm{lg}}_{e-\alpha }\right)+1\right)}^{1/\gamma }$
Generalised Logit (GL)	*f*(*x*) = 1/(1+exp(-*α*-*β*lg(*x*)))^γ^	$x=10^{\left(\left(-\mathrm{lg}\left({\left(\frac{1}{Y}\right)}^{\frac{1}{\gamma }}-1\right)/{\mathrm{lg}}_{e-\alpha }\right)/\beta \right)}$


$$R^{2}=1-\frac{\sum_{i=1}^{n}{\left(y_{i}-{\hat{y}}_{1}\right)}^{2}}{\sum_{i=1}^{n}{\left(y_{i}-\bar{y}\right)}^{2}}$$ (4)

$$\text{MSE}=\sqrt{\frac{1}{n}\sum_{i=1}^{n}{\left(y_{i}-{\hat{y}}_{l}\right)}^{2}}$$ (5)

$$\text{MAE}=\frac{\sum |y-\hat{y}|}{\text{n}}$$ (6)

In the formula, n represents the number of samples, *y*_*i*_ represents the true value, $\hat{y_{l}}$ represents the predicted value, and $\bar{y}$ represents the mean of the true values.

In the CRC fitting process, an additional observation confidence interval (OCI) was incorporated. This OCI accounts for both the uncertainty of the raw observation data and the uncertainty associated with fitting the nonlinear function to the experimental data [49].


$$OCI=\hat{y}\pm t_{\left(n-m,\frac{a}{2}\right)}\cdot \sqrt{s^{2}+vCv^{T}}$$ (7)

In the equation, α represents the level of significance. t represents the critical value of degrees of freedom and under α. C is the covariance matrix of parameter estimates obtained through nonlinear fitting. v is the row vector and v^*T*^ is the column vector.

## 3. Results and discussion

### 3.1 Results of single acute toxicity experiments

The results of the single acute toxicity experiments with Cr or Ni showed that the water parameters were stable before and after the experiment and suitable for the survival of ostracods, meeting the stability requirements for toxicological biological materials (Table 4).

**Table 4:** Water parameters of the solution before and after the *Heterocypris* sp. experiment.

Element	Group	Testing time	pH	Temperature °C	Dissolved oxygen	Hardness
					mg/L	mg/L
Cr	1	Before the experiment (0 h)	7.6 ± 0.1	25 ± 0.2	5.1 ± 0.2	155 ± 1
		After the experiment (96 h)	7.4 ± 0.1	25 ± 0.1	4.6 ± 0.2	155 ± 1
	2	Before the experiment (0 h)	7.6 ± 0.1	25 ± 0.2	5.0 ± 0.2	155 ± 1
		After the experiment (96 h)	7.5 ± 0.1	25 ± 0.1	4.5 ± 0.2	155 ± 1
	3	Before the experiment (0 h)	7.6 ± 0.1	25 ± 0.2	5.0 ± 0.2	155 ± 1
		After the experiment (96 h)	7.5 ± 0.1	25 ± 0.1	4.5 ± 0.2	155 ± 1
	4	Before the experiment (0 h)	7.6 ± 0.1	25 ± 0.2	5.0 ± 0.2	155 ± 1
		After the experiment (96 h)	7.5 ± 0.1	25 ± 0.1	4.5 ± 0.2	155 ± 1
	5	Before the experiment (0 h)	7.5 ± 0.1	25 ± 0.2	5.1 ± 0.2	155 ± 1
		After the experiment (96 h)	7.4 ± 0.1	25 ± 0.1	4.6 ± 0.2	155 ± 1
Ni	1	Before the experiment (0 h)	7.6 ± 0.1	25 ± 0.2	5.3 ± 0.2	147 ± 1
		After the experiment (96 h)	7.5 ± 0.1	25 ± 0.1	4.7 ± 0.2	147 ± 1
	2	Before the experiment (0 h)	7.6 ± 0.1	25 ± 0.2	5.4 ± 0.2	147 ± 1
		After the experiment (96 h)	7.4 ± 0.1	25 ± 0.1	4.7 ± 0.2	147 ± 1
	3	Before the experiment (0 h)	7.6 ± 0.1	25 ± 0.2	5.4 ± 0.2	147 ± 1
		After the experiment (96 h)	7.3 ± 0.1	25 ± 0.1	4.4 ± 0.2	147 ± 1
	4	Before the experiment (0 h)	7.6 ± 0.1	25 ± 0.2	5.3 ± 0.2	147 ± 1
		After the experiment (96 h)	7.4 ± 0.1	25 ± 0.1	4.2 ± 0.2	147 ± 1
	5	Before the experiment (0 h)	7.6 ± 0.1	25 ± 0.2	5.3 ± 0.2	147 ± 1
		After the experiment (96 h)	7.5 ± 0.1	25 ± 0.1	4.1 ± 0.2	147 ± 1

Note: The value following this symbol (“±”) indicates the standard errors.

With the increase of Cr and Ni concentrations and the extension of the experimental time (0–96 h), the acute toxicity effects of Cr and Ni on *Heterocypris* sp. were significantly enhanced, and the mortality rate also increased significantly. For example, when the concentrations of Cr and Ni were 15 and 44 mg/L, the mortality rate of *Heterocypris* sp. reached 100% after 72 h (Table 5).

**Table 5:** Single acute toxicity experiment results of Cr and Ni on *Heterocypris* sp.

Element	Group	Mortality at different experimental times %			
		24h	48h	72h	96 h
Cr	1	3	13	20	23
	2	10	23	37	47
	3	20	33	43	53
	4	37	50	63	77
	5	67	83	100	100
Ni	1	7	13	23	37
	2	23	30	40	57
	3	30	43	67	90
	4	43	67	87	93
	5	67	83	100	100
Blank control group		0	0	0	0

The CRC fitting results of *Heterocypris* sp. at different Cr and Ni concentrations were satisfactory (R^2^ averaged 0.98, MAE averaged 0.03) (Table 6). The overall trend of the effect concentration values of LC_10_, LC_30_, and LC_50_ decreased with the extension of time. For example, the LC_50_ values of Cr for *Heterocypris* sp. at 24, 48, 72, and 96 h were 8.50, 3.92, 1.86, and 1.07 mg/L, respectively. The difference between the maximum and minimum was 7.42 mg/L, and the multiple difference was 8. The LC_50_ values of Ni for *Heterocypris* sp. at 24, 48, 72, and 96 h were 25.94, 13.37, 7.65, and 4.7 mg/L, respectively. They exhibited a maximum and minimum difference of 21.24 mg/L and a multiple difference of 6.

**Table 6:** CRC fitting dataset of single acute toxicity experiments on *Heterocypris* sp.

Element	Exposure time/h	Function	α	β	γ	R^2^	MAE	Effect concentration/mg/L		
								LC_10_	LC_30_	LC_50_
								(Confidence Interval)	(Confidence Interval)	(Confidence Interval)
Cr	24	GL	-5.91	4.31	0.34	0.99	0.01	0.63 (0.34–0.97)	3.59 (2.93–4.3)	8.5 (7.29–9.83)
	48	Weibull	-1.18	1.38	/	0.98	0.03	0.17 (0.03–0.42)	1.29 (0.71–2.1)	3.92 (2.66–5.64)
	72	Weibull	-0.72	1.3	/	0.94	0.06	0.07 (0.01–0.31)	0.58 (0.14–1.43)	1.86 (0.86–3.68)
	96	Weibull	-0.4	1.21	/	0.96	0.05	0.03 (0.01–0.12)	0.3 (0.10–0.66)	1.07 (0.59–1.86)
Ni	24	BCW	-3.13	0.88	-0.03	0.98	0.03	2.74 (0.38–8.47)	11.57 (5.64–19.52)	25.94 (16.06–39.99)
	48	BCW	-3.17	1.31	-0.16	0.99	0.01	2.1 (0.67–3.61)	6.54 (5.04–8.23)	13.37 (10.68–16.66)
	72	Weibull	-2.48	2.39	/	0.99	0.01	1.25 (0.96–1.54)	4.03 (3.61–4.47)	7.65 (7.12–8.2)
	96	GL	-9.44	8.94	0.2	0.98	0.023	0.59 (0.38–1.94)	2.42 (1.01–4.08)	4.7 (3.29–6.3)

Note: observation confidence interval is 95%.

**Fig 4:**
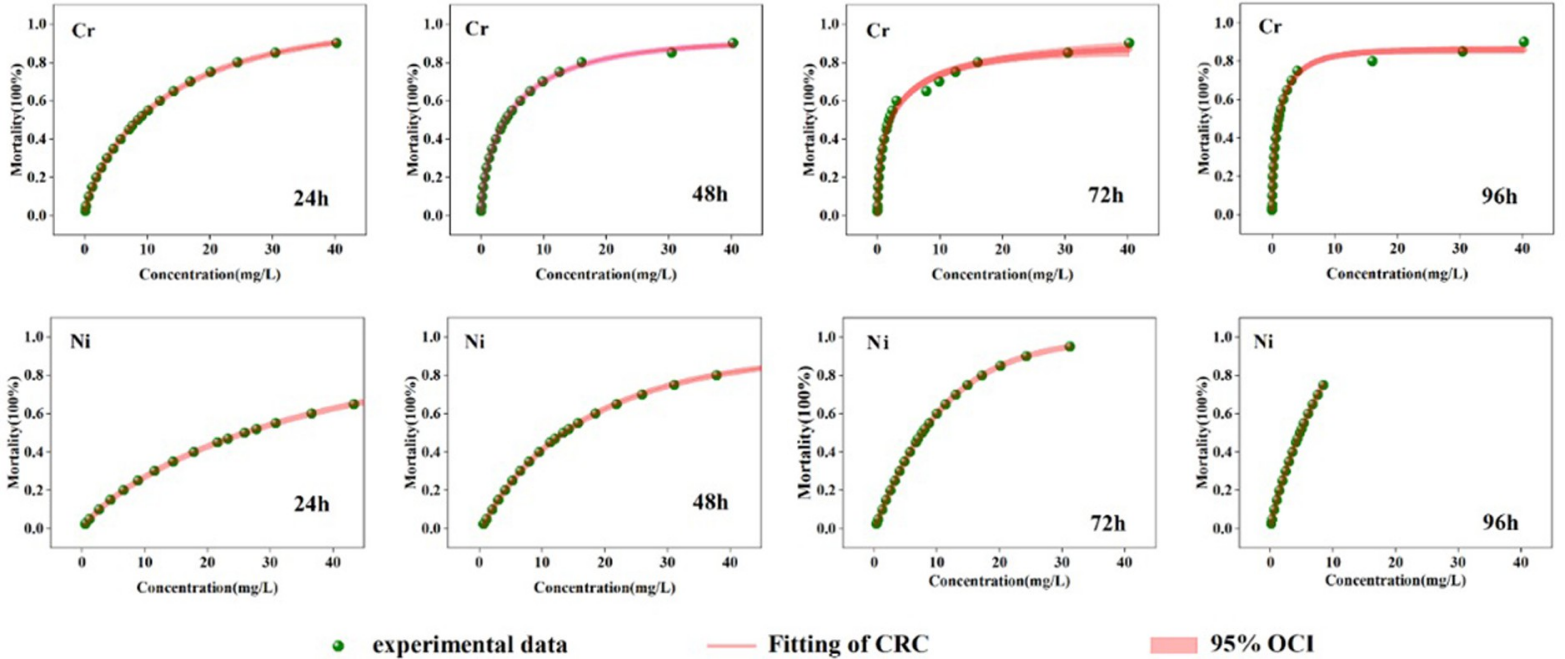
CRC fitting graph of single acute toxicity experiments of Cr and Ni on *Heterocypris* sp.

The CRC fitting of the single acute toxicity experiments of Cr and Ni showed acceptability, with small confidence intervals and high accuracy [50]. Therefore, the fitting curves were reliable. The fitting results showed a positive correlation between the concentration of Cr or Ni in the solution and the mortality rate of the organisms (Fig 4). However, the trend varied depending on factors such as time or concentration. For example, as exposure time increased, the toxicity of Cr to *Heterocypris* sp. rose, leading to a sharp rise in mortality rate. Furthermore, the rate of increase appeared to plateau after 96 hours. Additionally, the sensitivity of *Heterocypris* sp. to Ni was initially low. After 72 h, the toxicity of Ni significantly increased, followed by a sharp rise in the mortality rate. Therefore, the mortality rate of test organisms exhibited a significant time- and dose-dependent response to Cr and Ni concentrations in the solution.

### 3.2 Results of the Cr-Ni combined acute toxicity experiment

**Fig 5:**
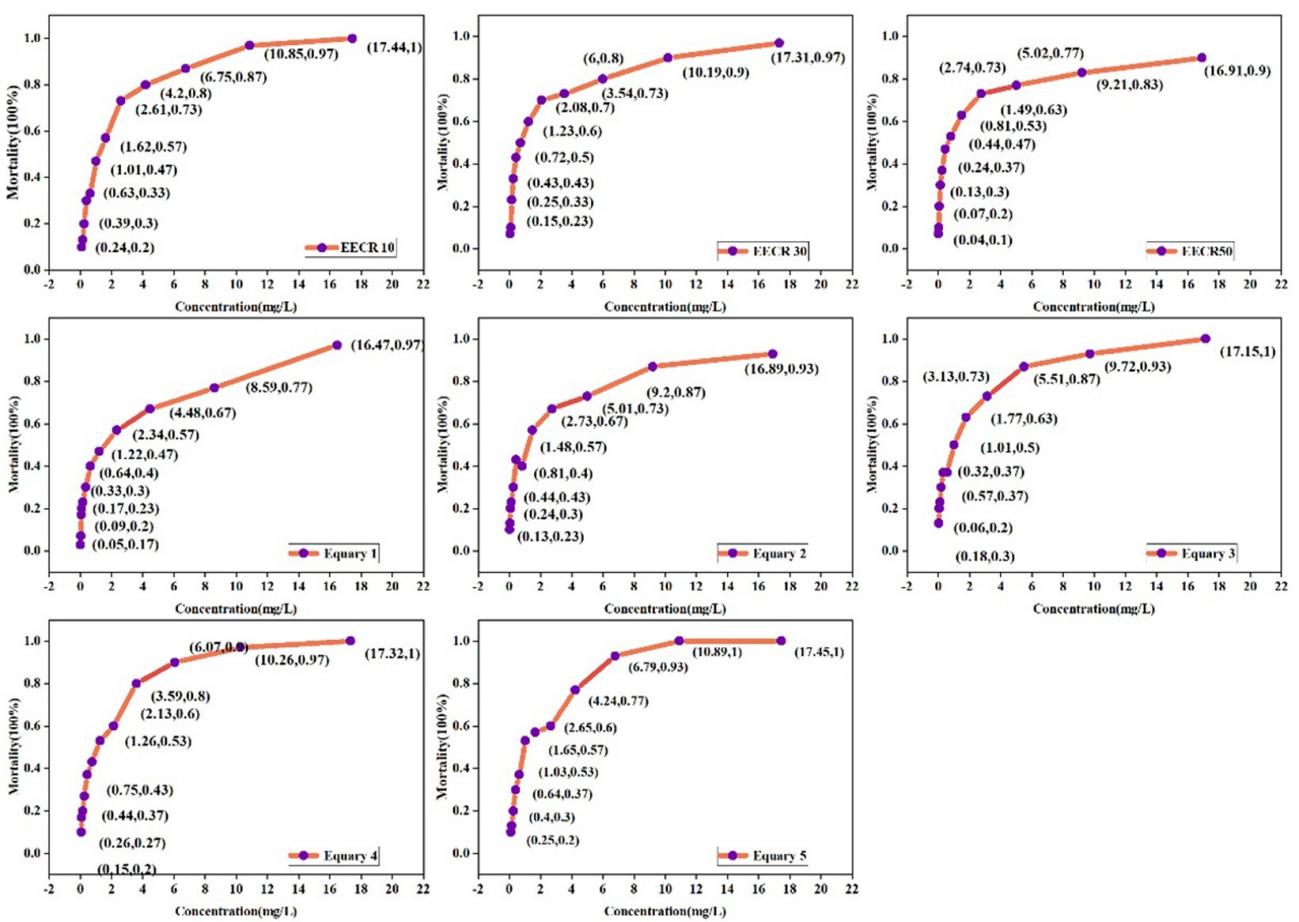
96 h combined acute toxicity experiment results of Cr-Ni on Heterocypris sp.

The combined toxicity of Cr and Ni depended on the concentration levels and relative ratios of the mixture. As the Equary and EECR concentration ratios increased below 4 mg/L, the toxicity to *Heterocypris* sp. increased, leading to a sharp rise in mortality rate (Fig 5). After 8 mg/L, it tended to be flat. *Heterocypris* sp. exhibited greater sensitivity to the Equary mixed concentration ratio. The mortality rate reached near 100% at concentrations exceeding 10 mg/L.

As the mixture concentration increased, the LC_50_ of the EECR combination gradually decreased (Table 7). This decrease in LC_50_ indicated that the toxicity of the mixture increased. The EECR 50 group exhibited an LC_50_ value of 0.56 mg/L. The maximum and minimum LC50 values within the EECR groups differed by a factor of 2. The LC_50_ of the EquRay combination decreased as the Ni concentration increased, and the toxicity gradually increased. The EquRay 5 group had an LC_50_ value of 0.74 mg/L. The maximum and minimum LC_50_ values within the EquRay groups differed by a factor of 2.

**Table 7:** Comparison of LC_30_ and LC_50_ data from the 96 h combined acute toxicity experiment of Cr-Ni and QSAR model predictions on *Heterocypris* sp.

Combination	LC_30_ (Effect concentration/mg/L)		LC_50_ (Effect concentration/mg/L)	
	Measured value (Confidence interval)	QSAR	Measured value (Confidence interval)	QSAR
EECR 10	0.46 (0.41–0.52)	0.46	1.07 (1.02–1.17)	1.17
EECR 30	0.24 (0.20–0.29)	0.24	0.66 (0.55–0.78)	0.66
EECR 50	0.16 (0.14–0.18)	0.16	0.56 (0.49–0.63)	0.56
EquRay 1	0.46 (0.35–0.59)	0.46	1.48 (1.15–1.87)	1.47
EquRay 2	0.36 (0.26–0.47)	0.35	1.25 (1.01–1.55)	1.25
EquRay 3	0.24 (0.19–0.3)	0.23	1.03 (0.88–1.19)	1.11
EquRay 4	0.24 (0.28–0.32)	0.22	0.97 (0.86–1.1)	0.97
EquRay 5	0.22 (0.18–0.27)	0.22	0.74 (0.68–0.62)	0.74

**Fig 6:**
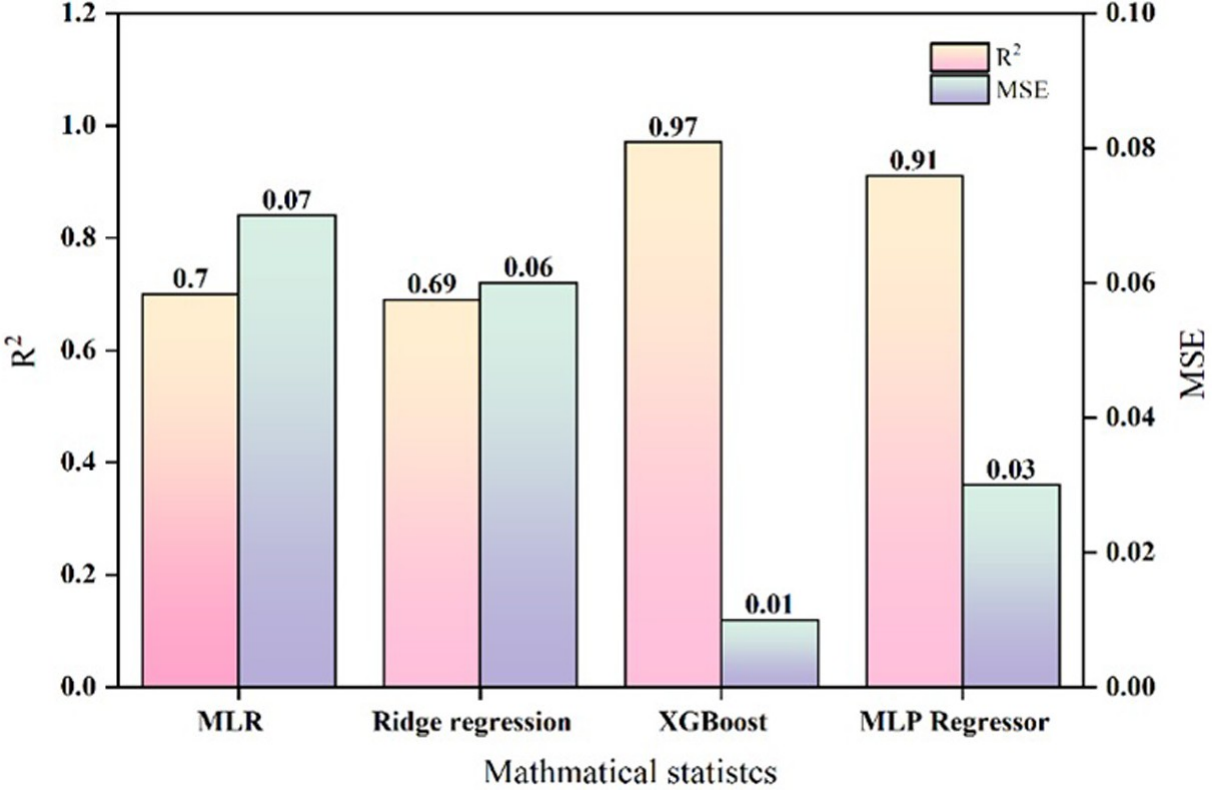
Mathematical statistics regression results chart of combined acute toxicity experiment.

**Fig 7:**
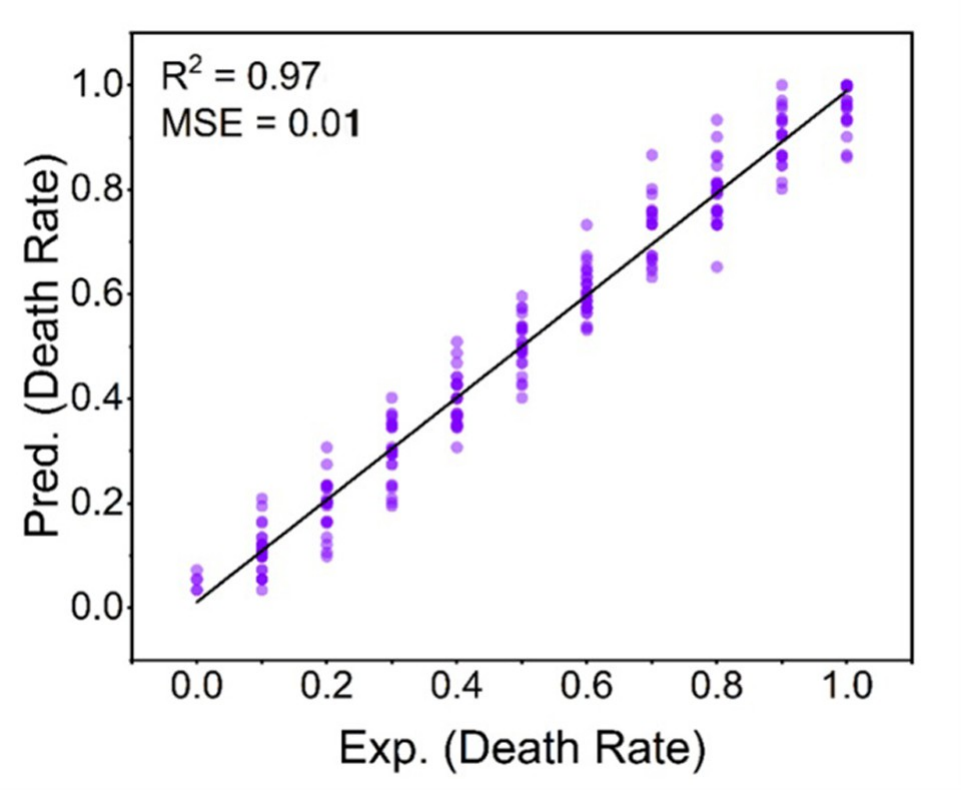
QSAR prediction results chart of combined acute toxicity experiment.

Different statistical methods were used to regress the experimental dataset (Fig 6). The best result was obtained using XGBoost, with an R^2^ value of 0.97 and MSE of 0.01. This result was suitable for building a QSAR model. Fig 7 showed the predicted results of the QSAR model for the combined toxicity experiment. The model had excellent predictive performance and was highly consistent with the experimental results.

## 3.3 Discussion

### 3.3.1 Single acute toxicity of Cr and Ni to *Heterocypris* sp

*Heterocypris* sp. exhibited the following symptoms of poisoning in high concentrations of Cr and Ni solutions: abnormal movement, loss of free swimming ability, sinking to the bottom of the container, and finally cardiac arrest and death. The toxicity mechanism of heavy metals can be roughly divided into three aspects [51]. First, heavy metals were adsorbed on the surface of organs, affecting the normal physiological functions of the organs. Second, heavy metals could induce cellular deformities, leading to cell apoptosis. Third, heavy metals could damage the structure and activity of DNA, enzyme functional proteins, etc., causing damage to genetic and metabolic functions. In the single acute toxicity experiments, Cr and Ni both induced oxidative stress and cell apoptosis [52]. Their toxicity mechanisms were related to MAPKs (mitogen-activated protein kinases), which were highly toxic. For example, 0.2 mg/L of Cr could cause toxicity.

The 96 h-LC_50_ values of Cr and Ni for *Heterocypris* sp. are 1.07 mg/L and 4.7 mg/L, respectively (Table 8). According to the calculated toxicity factor (the ratio of 96 h-LC_50_ values) [30], Cr is 4 times more toxic than Ni. The results are consistent with previous research by Fargasova et al. on the toxicity of Cr to freshwater organisms. For example, Fargasova et al. [56, 57] studied the toxicity of Cr and Ni to *Daphnia magna*, and found that Cr was about 4 times more toxic than Ni. Compared to other aquatic organisms, *Heterocypris* sp. exhibits a higher sensitivity to Cr and Ni.

**Table 8:** 96 h-LC_50_ of Cr and Ni single acute toxicity to different organisms.

Phylum	Species	Cr	Ni	Reference
Annelidan	*Aeolosoma headleyi*	1.88 mg/L		[53]
Protoceratophora	*Philodina roseola*	4.4 mg/L		[54]
Arthropoda	*Heterocypris* sp.	1.07 mg/L	4.7 mg/L	present study
	*Macrobrachium nipponen*	2.24 mg/L		[55]
	*Daphnia magna*	0.97 mg/L	4.08 mg/L	[56, 57]
Chordata, phylum of Chordata	*Oncorhynchus mykiss (Juvenile fish)*	4.4 mg/L		[58]
	*Pimephales promelas (Juvenile fish)*		4.58 mg/L	[59]
	*Cyprinus carpio(Juvenile fish)*		6.9 mg/L	[60]
	*Lepomis gibbosus*		8 mg/L	[61]

According to the Global Harmonized System of Classification and Labelling of Chemicals (GHS) [62] for acute aquatic toxicity of chemicals, if 1 < 96 h-LC_50_ < 10, it is considered to be highly toxic. Cr and Ni have both reached high toxicity.

#### 3.3.2 Combined acute toxicity of Cr and Ni to *Heterocypris* sp

When evaluating the combined acute toxicity of Cr with EquRay 1 and 2 (Table 9), the antagonistic effect was observed. This means the mixtures were less toxic than Cr alone, as indicated by the values (SR values) being below 1. In the remaining combinations, Cr and Ni showed synergistic effects (SR > 1), indicating that the toxicity of the mixed solutions was higher than the toxicity of either Cr or Ni alone. The antagonistic effect between the two metals may be due to the fact that the metals in the solution have similar binding affinities to the cell surface, and one metal reduces the available binding sites for another metal [63]. Since the binding affinity of Ni to the cell surface is slightly higher than that of Cr, it will preferentially bind to the cell surface, resulting in Ni occupying more binding sites and reducing the effective binding sites of Cr, thereby reducing its toxicity [64]. Verriopoulos et al. [65] found in their ecological toxicological study of Cr and Ni on *Tisbe holothuriae* that high concentrations of Cr reduced the combined toxicity of Cr-Ni. This result is similar to that of EquRay 1 and EquRay 2 in the Cr-Ni combination experiments.

**Table 9:** Qualitative evaluation of Cr-Ni combined acute toxicity on *Heterocypris* sp.

Combination	Cr-Ni		
	LC_50_	SR(Cr)	SR(Ni)
EECR 10	1.07	1.01	4.41
EECR 30	0.66	1.63	7.16
EECR 50	0.56	1.93	8.45
EquRay 1	1.48	0.73	3.19
EquRay 2	1.25	0.86	3.75
EquRay 3	1.03	1.04	4.57
EquRay 4	0.97	1.1	4.84
EquRay 5	0.74	1.45	6.37

In combined toxicity experiments, the concentrations and proportions of Cr and Ni affect the combined toxicity. When combined, the ratio of individual metal concentrations can influence the overall toxicity of the mixture. For example, in the Cr-Ni mixture experiments of EquRay 1 and EquRay 2 for *Heterocypris* sp., the combined toxicity increased by 1.2 times, when the Cr:Ni concentration ratio increased from 5:1 to 4:2. This toxic rule has a certain correlation with the magnitude of the single toxicity of Cr and Ni. The combined toxicity of Cr-Ni mixed solutions is higher than the single toxicity of Ni, but lower than the single toxicity of Cr.

#### 3.3.3 Combined acute toxicity model of Cr and Ni to *Heterocypris* sp

**Fig 8:**
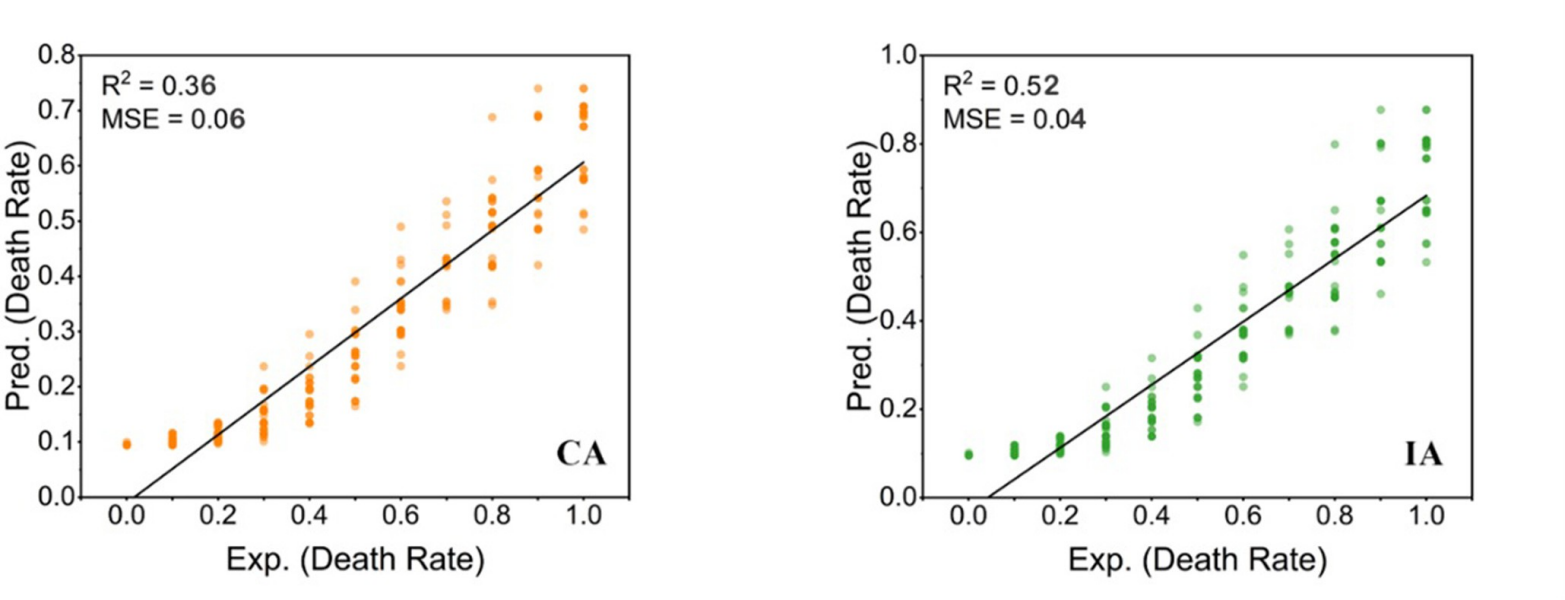
CA and IA predicted results charts of Cr-Ni combined acute toxicity experiment.

This study compared the accuracy of a QSAR model with CA and IA models (Figs 7 and 8). The QSAR model achieved significantly higher performance, with an R^2^ of 0.97 and MSE of 0.01, compared to CA (R^2^: 0.36, MSE: 0.06) and IA (R^2^: 0.52, MSE: 0.04). Furthermore, the QSAR model predictions closely matched the experimental results for *Heterocypris* sp.

## 4. Conclusions

Heavy metal pollution caused by mining has attracted widespread attention. This study investigated the ecotoxicological effects of common heavy metal pollution (Cr and Ni) in mines on ostracods (*Heterocypris* sp.). The acute toxic effects of Cr and Ni on *Heterocypris* sp. were both linearly positively correlated with the dose. The relationship between time and effect was evident, with longer experimental durations resulting in lower LC_50_ values, indicating higher toxicity. In a single acute toxicity test, Cr was more toxic to *Heterocypris* sp. than Ni, with 96 h-LC_50_ values of 1.07 mg/L (Cr) and 4.7 mg/L (Ni), respectively. In combined toxicity, the combined toxicity pattern of Cr-Ni was related to the magnitude of the single toxicity of Cr and Ni, and could produce antagonistic and synergistic effects. For example, high concentrations of Cr could reduce the combined toxicity of Cr-Ni, resulting in an antagonistic effect. In other words, the combined toxicity of the Cr-Ni mixture was higher than the single toxicity of Ni but lower than the single toxicity of Cr. The results of the QSAR model prediction of the combined toxicity of Cr-Ni were found to be highly consistent with the results of the combined acute toxicity experiment of *Heterocypris* sp., compared with the prediction results of the CA and IA models. Therefore, the QSAR model has accurate combined toxicity prediction ability and can be used to predict the toxicity of heavy metal pollutants in mine wastewater.

## Supporting information

S1 Table Results of a 96-hour combined acute toxicity experiment of Cr-Ni on *Heterocypris* sp. (DOC)
