# Supplementary material for: Identification of the single and combined acute toxicity of Cr and Ni with Heterocypris sp. and the quantitative structure-activity relationship (QSAR) model
Source: PLoS One. 2024 Mar 21;19(3):e0300800. doi: 10.1371/journal.pone.0300800 (PMC10957083; doi:10.1371/journal.pone.0300800)
Supplement: S1 Table — (DOC) [file pone.0300800.s001.doc]

**S1 Table.** Results of a 96-hour combined acute toxicity experiment of Cr-Ni on *Heterocypris* sp.

| Concentration design combination | Concentration ratio (100%) | | Total Concentration  mg/L | Mortality rate  % |
| --- | --- | --- | --- | --- |
|  | K_2_Cr_2_O_7_ | Ni(NO_3_)_2_ |  |  |
| EECR 10 | 0.05 | 0.95 | 17.44 | 100 |
|  |  |  | 10.85 | 97 |
|  |  |  | 6.75 | 87 |
|  |  |  | 4.20 | 80 |
|  |  |  | 2.61 | 73 |
|  |  |  | 1.62 | 57 |
|  |  |  | 1.01 | 47 |
|  |  |  | 0.63 | 33 |
|  |  |  | 0.39 | 30 |
|  |  |  | 0.24 | 20 |
|  |  |  | 0.15 | 13 |
|  |  |  | 0.09 | 10 |
| EECR 30 | 0.08 | 0.92 | 17.31 | 97 |
|  |  |  | 10.19 | 90 |
|  |  |  | 6.00 | 80 |
|  |  |  | 3.54 | 73 |
|  |  |  | 2.08 | 70 |
|  |  |  | 1.23 | 60 |
|  |  |  | 0.72 | 50 |
|  |  |  | 0.43 | 43 |
|  |  |  | 0.25 | 33 |
|  |  |  | 0.15 | 23 |
|  |  |  | 0.09 | 10 |
|  |  |  | 0.05 | 7 |
| EECR 50 | 0.11 | 0.89 | 16.91 | 90 |
|  |  |  | 9.21 | 83 |
|  |  |  | 5.02 | 77 |
|  |  |  | 2.74 | 73 |
|  |  |  | 1.49 | 63 |
|  |  |  | 0.81 | 53 |
|  |  |  | 0.44 | 47 |
|  |  |  | 0.24 | 37 |
|  |  |  | 0.13 | 30 |
|  |  |  | 0.07 | 20 |
|  |  |  | 0.04 | 10 |
|  |  |  | 0.02 | 7 |
| EquRay 1 | 0.53 | 0.47 | 16.47 | 97 |
|  |  |  | 8.59 | 77 |
|  |  |  | 4.48 | 67 |
|  |  |  | 2.34 | 57 |
|  |  |  | 1.22 | 47 |
|  |  |  | 0.64 | 40 |
|  |  |  | 0.33 | 30 |
|  |  |  | 0.17 | 23 |
|  |  |  | 0.09 | 20 |
|  |  |  | 0.05 | 17 |
|  |  |  | 0.03 | 7 |
|  |  |  | 0.02 | 3 |
| EquRay 2 | 0.31 | 0.69 | 16.89 | 93 |
|  |  |  | 9.20 | 87 |
|  |  |  | 5.01 | 73 |
|  |  |  | 2.73 | 67 |
|  |  |  | 1.48 | 57 |
|  |  |  | 0.81 | 40 |
|  |  |  | 0.44 | 43 |
|  |  |  | 0.24 | 30 |
|  |  |  | 0.13 | 23 |
|  |  |  | 0.07 | 20 |
|  |  |  | 0.04 | 13 |
|  |  |  | 0.02 | 10 |
| EquRay 3 | 0.19 | 0.81 | 17.15 | 100 |
|  |  |  | 9.72 | 93 |
|  |  |  | 5.51 | 87 |
|  |  |  | 3.13 | 73 |
|  |  |  | 1.77 | 63 |
|  |  |  | 1.01 | 50 |
|  |  |  | 0.57 | 37 |
|  |  |  | 0.32 | 37 |
|  |  |  | 0.18 | 30 |
|  |  |  | 0.10 | 23 |
|  |  |  | 0.06 | 20 |
|  |  |  | 0.04 | 13 |
| EquRay 4 | 0.10 | 0.90 | 17.32 | 100 |
|  |  |  | 10.26 | 97 |
|  |  |  | 6.07 | 90 |
|  |  |  | 3.59 | 80 |
|  |  |  | 2.13 | 60 |
|  |  |  | 1.26 | 53 |
|  |  |  | 0.75 | 43 |
|  |  |  | 0.44 | 37 |
|  |  |  | 0.26 | 27 |
|  |  |  | 0.15 | 20 |
|  |  |  | 0.09 | 17 |
|  |  |  | 0.05 | 10 |
| EquRay 5 | 0.04 | 0.96 | 17.45 | 100 |
|  |  |  | 10.89 | 100 |
|  |  |  | 6.79 | 93 |
|  |  |  | 4.24 | 77 |
|  |  |  | 2.65 | 60 |
|  |  |  | 1.65 | 57 |
|  |  |  | 1.03 | 53 |
|  |  |  | 0.64 | 37 |
|  |  |  | 0.40 | 30 |
|  |  |  | 0.25 | 20 |
|  |  |  | 0.16 | 13 |
|  |  |  | 0.10 | 10 |
